# Identification of a self‐sufficient cytochrome P450 monooxygenase from *Cupriavidus pinatubonensis* JMP134 involved in 2‐hydroxyphenylacetic acid catabolism, via homogentisate pathway

**DOI:** 10.1111/1751-7915.13865

**Published:** 2021-06-22

**Authors:** Raúl A. Donoso, Daniela Ruiz, Carla Gárate‐Castro, Pamela Villegas, José Eduardo González‐Pastor, Víctor de Lorenzo, Bernardo González, Danilo Pérez‐Pantoja

**Affiliations:** ^1^ Programa Institucional de Fomento a la Investigación, Desarrollo e Innovación (PIDi) Universidad Tecnológica Metropolitana Santiago Chile; ^2^ Center of Applied Ecology and Sustainability (CAPES) Santiago Chile; ^3^ Facultad de Ingeniería y Ciencias Universidad Adolfo Ibáñez Santiago Chile; ^4^ Laboratory of Molecular Adaptation, Department of Molecular Evolution Centro de Astrobiología (CSIC‐INTA) Madrid Spain; ^5^ Systems and Synthetic Biology Department Centro Nacional de Biotecnología (CNB‐CSIC) Campus de Cantoblanco Madrid Spain

## Abstract

The self‐sufficient cytochrome P450 RhF and its homologues belonging to the CYP116B subfamily have attracted considerable attention due to the potential for biotechnological applications based in their ability to catalyse an array of challenging oxidative reactions without requiring additional protein partners. In this work, we showed for the first time that a CYP116B self‐sufficient cytochrome P450 encoded by the *ohpA* gene harboured by *Cupriavidus pinatubonensis* JMP134, a β‐proteobacterium model for biodegradative pathways, catalyses the conversion of 2‐hydroxyphenylacetic acid (2‐HPA) into homogentisate. Mutational analysis and HPLC metabolite detection in strain JMP134 showed that 2‐HPA is degraded through the well‐known homogentisate pathway requiring a 2‐HPA 5‐hydroxylase activity provided by OhpA, which was additionally supported by heterologous expression and enzyme assays. The *ohpA* gene belongs to an operon including also *ohpT,* coding for a substrate‐binding subunit of a putative transporter, whose expression is driven by an inducible promoter responsive to 2‐HPA in presence of a predicted OhpR transcriptional regulator. OhpA homologues can be found in several genera belonging to Actinobacteria and α‐, β‐ and γ‐proteobacteria lineages indicating a widespread distribution of 2‐HPA catabolism via homogentisate route. These results provide first time evidence for the natural function of members of the CYP116B self‐sufficient oxygenases and represent a significant input to support novel kinetic and structural studies to develop cytochrome P450‐based biocatalytic processes.

## Graphical abstract

A self‐sufficient cytochrome P450 monooxygenase coded by *ohpA* gene is involved in 2‐hydroxyphenylacetate (2‐HPA) biodegradation, via homogentisate pathway in *Cupriavidus pinatubonensis* JMP134. The *ohpA* gene belongs to an operon whose expression is driven by an inducible promoter responsive to 2‐HPA. OhpA homologues can be found in several bacterial lineages suggesting a widespread distribution of 2‐HPA catabolism via homogentisate route.

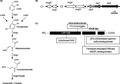

## Introduction

Cytochrome P450 enzymes are a large superfamily of cysteine thiolate‐coordinated b‐type haem proteins that bind oxygen generating highly reactive iron‐oxo species, which frequently catalyse reactions of monooxygenation, incorporating one oxygen atom to the substrate while the other oxygen atom is reduced to H_2_O (Denisov *et al*., 2005; Hrycay and Bandiera, 2015). These enzymes are broadly distributed in all life kingdoms having the ability to catalyse a vast number of chemically challenging reactions, making them powerful biocatalytic tools for biotechnology and synthetic biology (Bernhardt and Urlacher, 2014; Urlacher and Girhard, 2019). Cytochromes P450 act on a wide range of structurally different substrates, such as fatty acids, alkanes, steroids, vitamins, antibiotics, and diverse xenobiotics and drugs, playing key roles in biosynthesis and detoxification of a huge array of compounds (Van Bogaert *et al*., 2011; Peschke *et al*., 2016; Bhattacharya and Yadav, 2018; Ichinose and Kitaoka, 2018; Felpeto‐Santero *et al*., 2019; Klenk *et al*., 2019). Electron equivalents required in cytochrome P450 reactions are usually derived from NAD(P)H through an electron transfer chain represented by two main class of redox partners (Munro *et al*., 2007; McLean *et al*., 2015). Class I systems are represented by the first discovered microbial cytochrome P450: The *Pseudomonas putida* camphor hydroxylase P450cam (CYP101A1), a three‐component camphor‐hydroxylating system able to transfer electrons from NADH through a FAD‐binding protein (putidaredoxin reductase) into the iron–sulphur (2Fe‐2S) cluster‐binding ferredoxin (putidaredoxin) to the cytochrome P450 haem iron for catalysis (Mueller *et al*., 1995). Class II systems, such as membrane‐bound mammalian hepatic two‐component P450 enzymes, are associated with a diflavin reductase redox partner (named cytochrome P450 reductase; Murataliev *et al*., 2004; Pandey and Flück, 2013). Remarkably, it has been demonstrated that P450 systems are wider than was initially supposed, including cytochromes P450 bypassing redox partners, P450 fusions with non‐electron transfer domains, and P450 fusions with several types of redox partner proteins, apparently facilitating efficient electron transport between protein domains (Munro *et al*., 2007; McLean *et al*., 2015). In this context, one of the most interesting systems due to its potential biotechnological applications is the P450‐redox partner fusion systems represented by the catalytically self‐sufficient P450 BM3 from *Bacillus megaterium,* that is a single‐polypeptide enzyme comprising haem‐, FAD‐ and FMN‐binding domains, in which a soluble P450 fatty acid hydroxylase belonging to CYP102A subfamily is fused to a NADPH‐dependent reductase (class II), displaying a high catalytic efficiency and a well‐coupled reaction (Munro *et al*., 2002). This enzyme has a rather limited substrate range beyond its putative natural substrate, and it has been extensively engineered to catalyse oxidation of non‐physiological substrates such as pharmaceutical metabolites, monosaccharides, alkanes, aromatic compounds, short‐chain fatty acids and steroids (Meinhold *et al*., 2006; Lewis *et al*., 2009; Kille *et al*., 2011; Di Nardo and Gilardi, 2012; Whitehouse *et al*., 2012; Munday *et al*., 2016; O'Hanlon *et al*., 2017). Moreover, precise physiological role of the enzyme is still debatable since P450 BM3 expression is not induced in *B. megaterium* by saturated straight‐chain fatty acids (Whitehouse *et al*., 2012). Another type of catalytically self‐sufficient P450 with a distinctive redox partner was identified in *Rhodococcus* sp. NCIMB 9784 (Roberts *et al*., 2002), in which a soluble P450 domain is fused at the C‐terminus to a FMN and 2Fe‐2S ferredoxin containing reductase domain that resembles phthalate dioxygenase reductase (PDOR) from *Burkholderia cepacia* (Gassner *et al*., 1995; Chang and Zylstra, 1998). This clearly distinct *Rhodococcus* cytochrome was termed P450 RhF being classified in the CYP116B subfamily (De Mot and Parret, 2002). Close homologues of P450 RhF have been found in several species non‐related to Actinobacteria as the metal‐tolerant bacterium *Cupriavidus metallidurans* CH34 (Warman *et al*., 2012), the marine bacterium *Labrenzia aggregata* IAM 12614 (Yin *et al*., 2013), the alkane‐degrading *Acinetobacter radioresistens* S13 (Minerdi *et al*., 2015) and the halophile *Halomonas* sp. NCIMB 172 (Porter *et al*., 2018), suggesting that CYP116B subfamily of P450s is present in phylogenetically distant genera. The P450 RhF and their homologues show an apparent high level of substrate promiscuity (but with low activity), catalysing a range of O‐dealkylations, aromatic hydroxylations, epoxidations and asymmetric sulphoxidations (O'Reilly *et al*., 2013). The self‐sufficient nature of P450 RhF along with its apparent broader substrate range highlights this enzyme as an outstanding starting template for directed evolution and promising alternative to P450 BM3 for protein engineering endeavours (O'Reilly *et al*., 2013). However, the identification of physiologically relevant substrates of P450 RhF and their homologues is still elusive, hampering further advances in structure‐function studies (Roberts *et al*., 2003; Hunter *et al*., 2005; Celik *et al*., 2006).

**Fig. 1:**
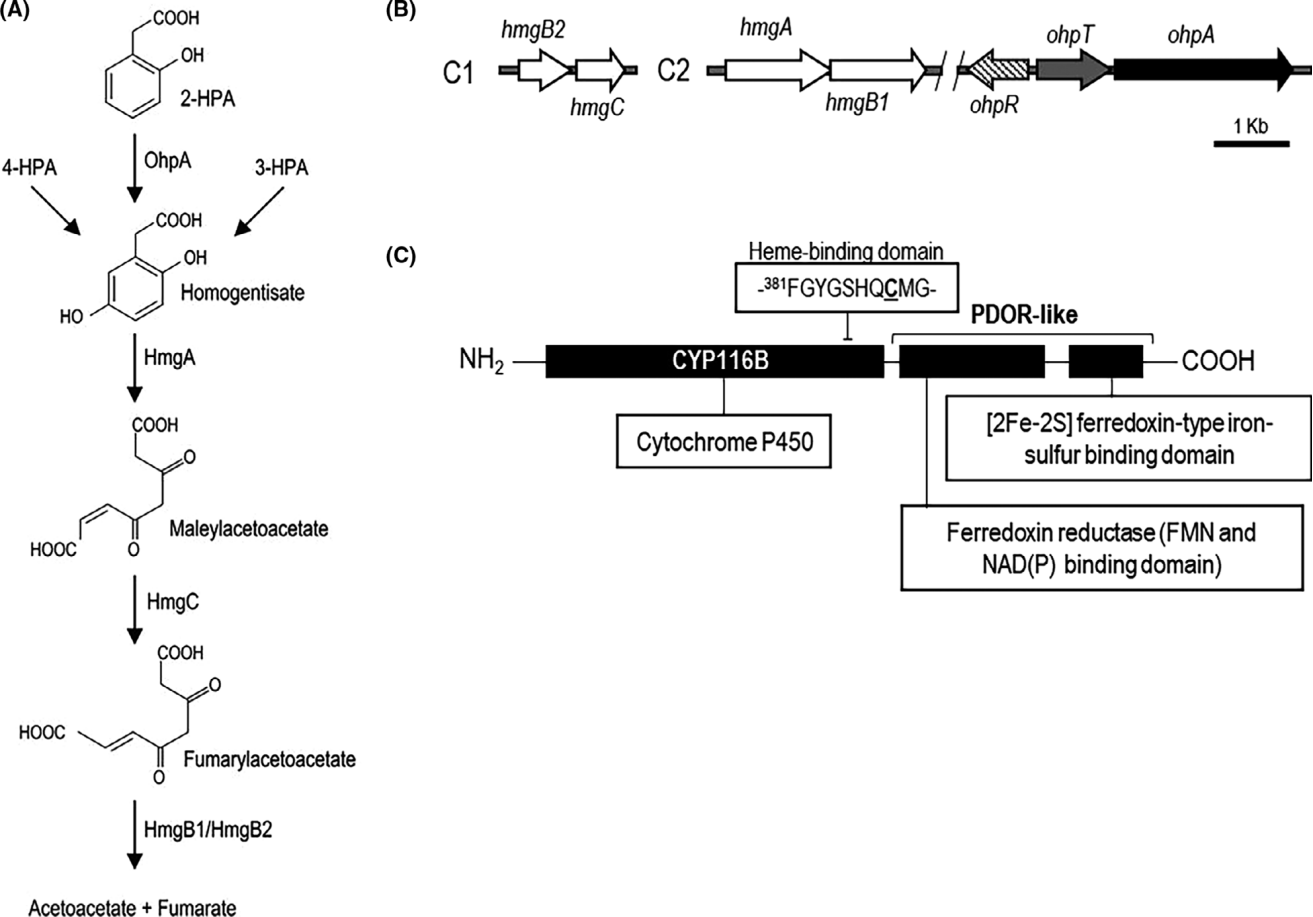
Putative 2‐hydroxyphenylacetate (2‐HPA) degradation pathway and gene clusters involved in 2‐HPA catabolism in *Cupriavidus pinatubonensis* JMP134. A. *C. pinatubonensis* JMP134 route channelling degradation of 2‐HPA, 3‐HPA and 4‐HPA through homogentisate producing acetoacetate and fumarate (adapted from Pérez‐Pantoja *et al*., 2008). B. Genes putatively involved in 2‐HPA/homogentisate catabolism in strain JMP134. C. Protein domain structures of self‐sufficient cytochrome P450 enzyme encoded by *ohpA* gene. The two fused domains are shown in bold. Specific functions are indicated in thin‐lines boxes. The protein residue ^388^Cysteine (haem‐iron proximal ligand) is underlined. C1: Main chromosome; C2: Secondary chromosome.

One bacterial strain in which a close homologue of P450 RhF can be identified is the versatile pollutant degrader *Cupriavidus pinatubonensis* JMP134 that uses a vast array of aromatic compounds as growth substrate based on the broad diversity of catabolic pathways encoded in its genome (Pérez‐Pantoja *et al*., 2008, 2012; Lykidis *et al*., 2010). However, a few degradation pathways used by strain JMP134 to metabolize some aromatic compounds such as 2‐hydroxyphenylacetate (2‐HPA) are still unknown (Pérez‐Pantoja *et al*., 2008). This substrate is a natural product found in plants belonging to genus *Astilbe* (Kindl and Billek, 1962; Kindl, 1969) and has been proposed as intermediate in phenylacetate catabolism through the well‐known homogentisate (2,5‐dihydroxyphenylacetate) pathway in *Aspergillus nidulans* (Mingot *et al*., 1999). As well, in an earlier report, it has been suggested that 2‐HPA could be metabolized via homogentisate in *P. fluorescens* ST (Baggi *et al*., 1983), for which it would be necessary an undiscovered 2‐hydroxyphenylacetate‐5‐hydroxylase enzyme. Conversely, the homogentisate‐ring cleavage pathway is a well‐reported metabolic route, widespread in all life domains because it is used for tyrosine catabolism, starting with the maleylacetoacetate‐producing enzyme homogentisate dioxygenase (HmgA) (Fig. 1A; Hildebrandt *et al*., 2015). This product is subsequently isomerized into fumarylacetoacetate by a GSH‐dependent enzyme (HmgC) and hydrolysed by a specific hydrolase (HmgB) to generate fumarate and acetoacetate channelled into tricarboxylic acid cycle (Fig. 1A).

Here, we provide genetic evidence that the physiological substrate of the self‐sufficient cytochrome P450 encoded in strain JMP134 is 2‐HPA. This substrate is transformed into homogentisate by a previously unnoticed 2‐HPA 5‐hydroxylase activity of this P450 RhF homologue, allowing its utilization as a sole carbon source in a way that is transcriptionally controlled by a 2‐HPA‐inducible promoter; and thus, revealing a landmark for catalytic and structural studies over this biotechnologically relevant enzyme family.

## Results and discussion

### 2‐HPA is degraded through homogentisate pathway in *C. pinatubonensis* JMP134

The metabolically versatile *C. pinatubonensis* JMP134 bacterium can grow on the *o*‐, *m*‐ and *p*‐ isomers of HPA as well as to well‐known substrates of the homogentisate‐ring cleavage pathway such as tyrosine and phenylalanine (Pérez‐Pantoja *et al*., 2008). In this strain, genes encoding homogentisate‐degrading enzymes are found distributed along its genome with *hmgA* and *hmgB1* encoded in a secondary chromosome, whereas *hmgC* and a second fumarylacetoacetate hydrolase‐encoding gene (hmgB2) located in the main chromosome (Fig. 1B). To get additional evidence for involvement of the homogentisate route in catabolism of all three HPA isomers, we conducted preliminary experiments on differential gene expression using a sub‐genomic DNA microarray of strain JMP134 and found a sharp induction of *hmgA* transcript levels as reported by median of ratios of spot signal intensities (values in brackets) in the presence of 1 mM 2‐HPA (23.1 ± 2.9), 3‐HPA (7.8 ± 1.5) and 4‐HPA (6.5 ± 1.2). These results prompted us to get additional and substantive evidence performing growth tests in 2‐, 3‐, 4‐HPA, tyrosine or phenylalanine as sole carbon and energy sources, with an *hmgA* mutant obtained by insertional inactivation. As expected, none of these substrates allowed growth of the *hmgA* derivative and liquid cultures turned yellow/brown colouring (see Fig. S1A, for 2‐HPA as example) suggesting accumulation and spontaneous polymerization of homogentisate, which produces melanin‐like compounds, such as pyomelanin, that confer this characteristic colour to the medium (Rodríguez‐Rojas *et al*., 2009; Han *et al*., 2015). Homogentisate accumulation in the *hmgA* mutant was confirmed by HPLC / UV detection in resting cells exposed to 2‐HPA (Fig. S1B). In contrast, the growth on phenylacetate (PA), a substrate not channelled through the homogentisate pathway (Pérez‐Pantoja *et al*., 2008, 2015) was not affected in the *hmgA* derivative (Fig. S1A). To confirm that the absence of a *hmgA* gene was responsible of the observed phenotype, the mutant strain was transformed with a plasmid expressing the *hmgA* gene controlled by the L‐arabinose‐inducible P_BAD_ promoter. This heterologous promoter was chosen due L‐arabinose is non‐toxic and is not a carbon source for *C. pinatubonensis* JMP134 allowing reliable growth tests in this strain (Donoso *et al*., 2011). This construct allowed restoration of growth abilities in all carbon sources channelled through homogentisate in *C. pinatubonensis* JMP134 (see Fig. S1A, including results for 2‐HPA as example). These results confirmed that 2‐HPA, and 3‐HPA and 4‐HPA, are channelled into the homogentisate pathway in strain JMP134, as previously proposed by Pérez‐Pantoja *et al*. (2008).

### A cytochrome P450‐coding gene was differentially expressed by strain JMP134 grown on 2‐HPA

**Fig. 2:**
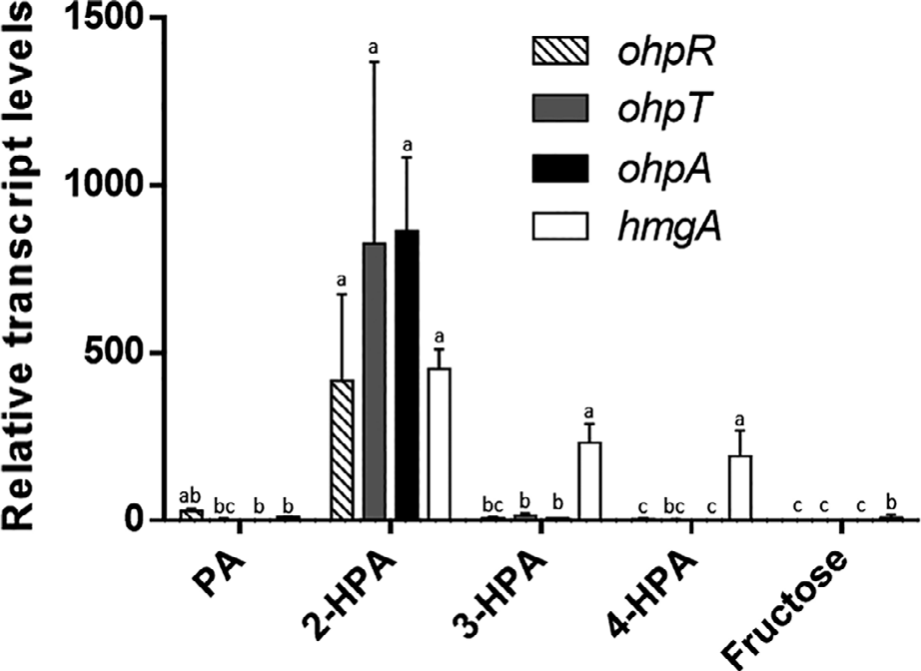
Transcript levels of putative 2‐hydroxyphenylacetate (2‐HPA) degradation genes from *Cupriavidus pinatubonensis* cells exposed to different phenylacetate compounds. Real‐time PCR analysis was performed for *ohpR*, *ohpT*, *ohpA* and *hmgA* genes expression in cells grown on phenylacetate (PA), 2‐HPA, 3‐HPA, 4‐HPA or fructose (control) as a sole carbon and energy sources. Transcript levels were normalized to the average value of transcript levels in fructose treatment. Additionally, 16S rRNA levels were used as a reference gene (internal control). All experiments were performed in three biological replicates. Error bars represent SEM. Different letters indicate statistically significant differences between treatments for each gene (one‐way analysis of variance, *P* < 0.05; Tukey’s test, *P* < 0.05), specifically in this graph the statistical group including 2‐HPA treatment (a) had a significantly much higher transcript levels as compared to any other groups (b, c, bc). The transcript levels of *hmgA* when cells were grown on 3‐HPA or 4‐HPA are also included in this statistical group (a).

Differential expression analyses performed with a sub‐genomic DNA microarray of *C. pinatubonensis* JMP134 also revealed a sharp increase in transcripts level (19.5 ± 3.7 as reported by median of ratios of spot signal intensities) of the gene labelled with locus tag Reut_B5278 in cultures exposed to 1 mM 2‐HPA, suggesting a role in biodegradation of this aromatic compound. This gene encodes a product with function not previously established but related to cytochrome P450, inferred by conserved domain analysis (Fig. 1C), pinpointing it to be a plausible candidate to encode a 2‐HPA 5‐hydroxylase activity responsible for homogentisate generation (accession IDs AAZ64624 or WP_011301389; termed *ohpA* by *ortho‐* hydroxyphenylacetate). To test if *ohpA* gene is involved in 2‐HPA catabolism, a quantitative real‐time PCR analysis of RNA extracted from mid‐log‐phase cells of *C. pinatubonensis* JMP134 growing on 2‐, 3‐, 4‐HPA, PA or fructose (a non‐related carbon source as basal level) was carried out. Transcript levels of the *ohpA* gene in 2‐HPA‐grown cells were at least two orders of magnitude higher than those of cells growing on any of the other compounds (Fig. 2), indicating a quite specific induction profile, and strongly suggesting its participation in 2‐HPA catabolism but not in degradation of 3‐ and 4‐HPA. In addition, quantitative analyses also showed that transcript levels of *hmgA* gene were clearly increased in 2‐HPA‐grown cells, like 3‐ and 4‐HPA‐grown cells but not in PA‐grown cells (Fig. 2), providing further evidence that catabolism of these three isomers involves the specific activation of the homogentisate‐ring cleavage pathway, but most probably recruiting different hydroxylases for channelling each one into this route (Fig. 1A).

### Mutational analyses, heterologous expression and enzyme assays confirm the key role of *ohpA* gene in biodegradation of 2‐HPA

**Fig. 3:**
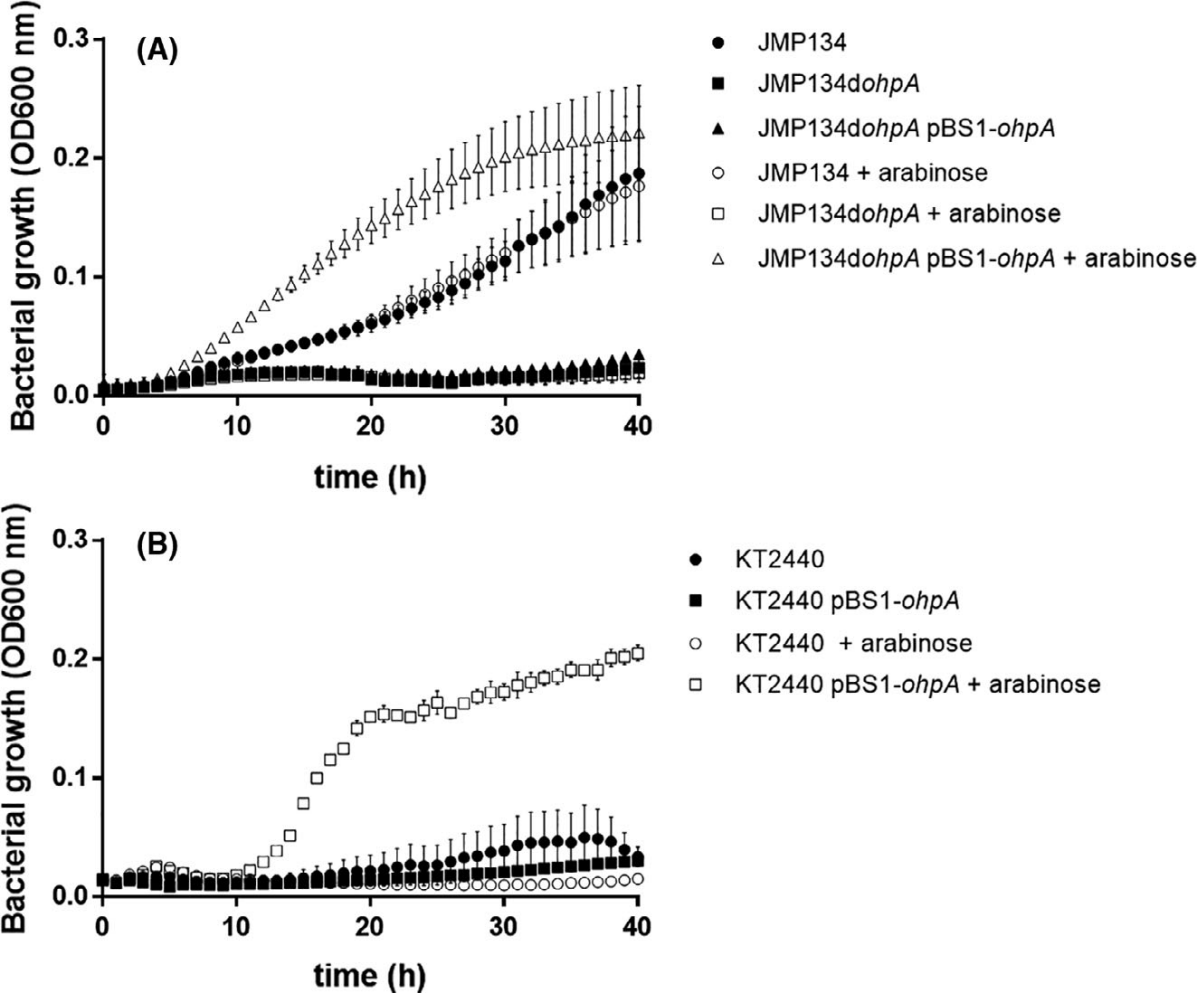
Growth on 2‐HPA of *Cupriavidus pinatubonensis* JMP134, *Pseudomonas putida* KT2440 and their derivatives. A. Growth of *C. pinatubonensis* wild type (JMP134), *ohpA* mutant (JMP134d*ohpA*) and *ohpA* mutant expressing *ohpA* gene driven by a heterologous P_BAD_ promoter (JMP134d*ohpA* pBS1‐*ohpA*), on 2‐HPA as a sole carbon and energy source, in the presence or absence of l‐arabinose. B. Growth of *P. putida* KT2440 and its derivative expressing *ohpA* gene driven by the heterologous P_BAD_ promoter (KT2440 pBS1‐*ohpA*), on 2‐HPA as a sole carbon and energy source, in the presence or absence of l‐arabinose. Three biological replicates were performed for growth measurements. Error bars indicate the standard deviation.

**Fig. 4:**
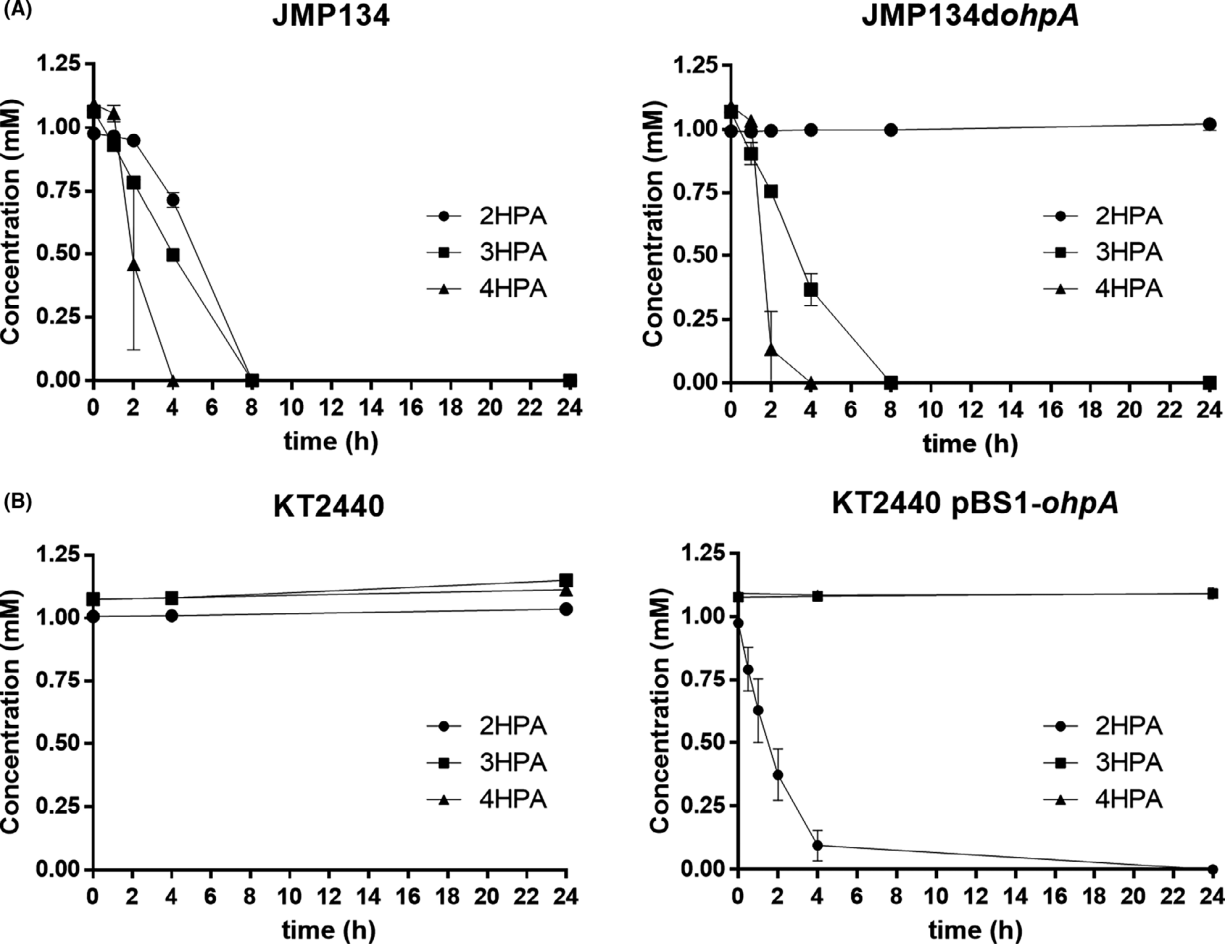
Resting cell assays of *Cupriavidus pinatubonensis* JMP134, *Pseudomonas putida* KT2440 and their derivatives in presence of hydroxyphenylacetate isomers. A. Resting cells of *C. pinatubonensis* JMP134, and the *ohpA* mutant (JMP134d*ohpA*) were grown on fructose 20 mM plus 2‐HPA 2.5 mM as inducer, washed and subsequently exposed to 1 mM 2‐HPA, 3‐HPA or 4‐HPA. B. Resting cells of *P. putida* KT2440, and the derivative expressing *ohpA* gene driven by the heterologous P_BAD_ promoter (KT2440 pBS1‐*ohpA*) were grown on succinate 30 mM plus arabinose 2.5 mM as inducer, washed and subsequently exposed to 1 mM 2‐HPA, 3‐HPA or 4‐HPA. Two biological replicates were performed for growth measurements. Error bars indicate the standard deviation.

As *ohpA* gene was strongly overexpressed during growth in 2‐HPA, we further explored the role of OhpA as a cytochrome P450 system performing a putative 2‐HPA 5‐hydroxylase activity. A derivative of *C. pinatubonensis* JMP134 harbouring a defective *ohpA* gene was generated through insertional inactivation. As expected, this derivative strain was unable to grow on 2‐HPA as a sole carbon and energy source (Fig. 3A) but was still able to proliferate in presence of 3‐ and 4‐HPA. Moreover, 2‐HPA was not transformed at all in resting cells assays of the mutant derivative, contrasting the consumption profile displayed by the wild‐type strain, conversely, 3‐ and 4‐HPA are consumed as the similar rate by the wild type and the mutant derivative strain (Fig. 4A). For further confirmation, this *ohpA* mutant was complemented with a plasmid construct containing *ohpA* gene driven by the l‐arabinose‐inducible P_BAD_ promoter. This derivative recovered the ability to grow on 2‐HPA only in presence of l‐arabinose (Fig. 3A), indicating that *ohpA* gene is essential for 2‐HPA degradation in strain JMP134. The higher growth rate on 2‐HPA displayed by the complemented derivative compared with the parent JMP134 strain would be indicative that the wild‐type expression level of *ohpA* is a limiting step in 2‐HPA catabolism. Then, to gain full certainty of the *ohpA* function the plasmid construct containing such gene was introduced into *P. putida* KT2440, a well‐known aromatic‐degrader bacterium that harbours homogentisate pathway but is unable to grow on 2‐HPA (Jiménez *et al*., 2002), and it is also unable to use L‐arabinose as carbon source (Wang *et al*., 2019). The presence of *ohpA* was sufficient to allow growth of strain KT2440 on 2‐HPA exclusively in presence of L‐arabinose (Fig. 3B), and a resting cells assay showed complete 2‐HPA consumption (and not for 3‐HPA or 4‐HPA) in this condition (Fig. 4B).

Finally, to indirectly evaluate OhpA enzyme specificity, we assayed the substrate‐dependent NADH oxidation in cell extracts of *P. putida* KT2440 harbouring pBS1‐*ohpA* when grown in presence of 5 mM l‐arabinose. The NADH oxidation activities assayed were 951.6 ± 40.7 U g^−1^, 43.9 ± 12.5 U g^−1^ and 31.4 ± 11.5 U g^−1^ when 2‐HPA, 3‐HPA or 4‐HPA were, respectively, added to the reaction mixture, suggesting that 2‐HPA is a strongly preferred substrate by OhpA. On the contrary, no measurable 2‐HPA‐dependent NADH oxidation activity was detected in a cell‐free extract of *P. putida* KT2440 lacking pBS1‐*ohpA*. Altogether, the results of genetic and enzyme assays unequivocally confirmed that OhpA enzyme is key in 2‐HPA catabolism.

### Expression of *ohpA* gene is controlled by an inducible promoter responsive to 2‐HPA

**Fig. 5:**
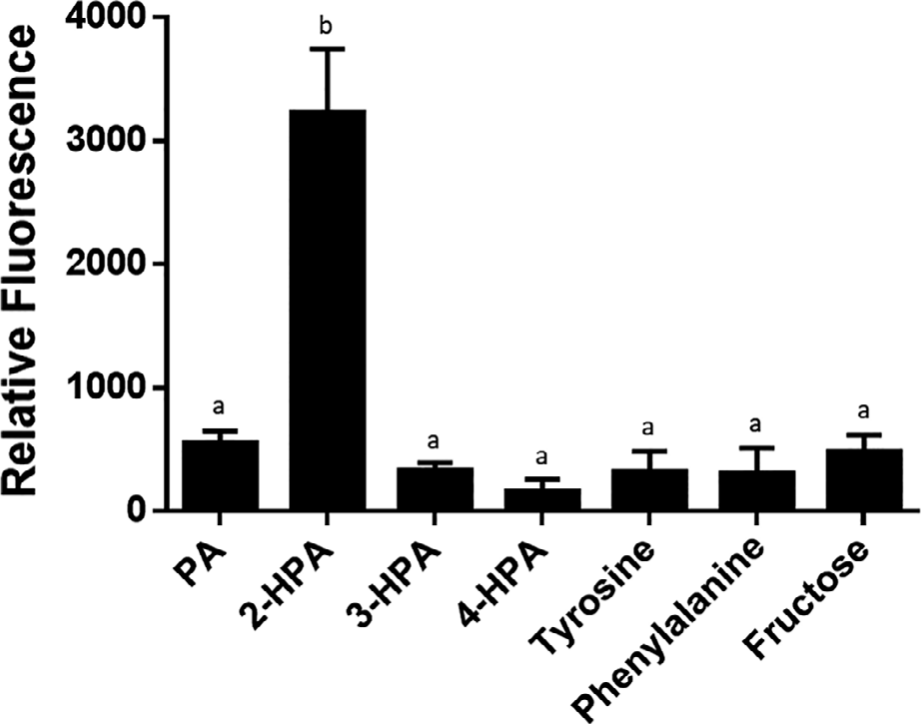
Activity of a GFP transcriptional reporter constructed with the intergenic *ohpR‐ohpT* region in presence of hydroxyphenylacetate isomers and related compounds metabolized by *Cupriavidus pinatubonensis* JMP134. Green fluorescent protein levels per cell biomass (relative fluorescence) obtained from the corresponding transcriptional fusion of *ohp* genes in strain JMP134 cells grown on 5 mM phenylacetate (PA), 2‐, 3‐, 4‐hydroxyphenylacetate (HPA), tyrosine, phenylalanine or fructose, after 24 h are shown. Error bars indicate the standard deviation. Different letters indicate statistically significant differences between treatments (one‐way analysis of variance, *P* < 0.05; Tukey's test, *P* < 0.05).

Visual inspection of the gene context of *ohpA* gene revealed an adjacent gene, located 46 bp upstream of *ohpA*, identified by conserved domain analysis as a putative tripartite tricarboxylate transporter substrate‐binding protein (Fig. 1B; *ohpT* gene) that belongs to the *Bordetella* uptake gene protein family (Antoine *et al*., 2003), suggesting a putative 2‐HPA transporter function. In addition, 130 bp upstream of the *ohpT* gene, in opposite orientation, a putative transcriptional regulator was found (Fig. 1B; *ohpR* gene), which may be involved in regulation of 2‐HPA catabolism. OhpR belongs to the IclR‐type family of regulators that can act as repressors, activators or proteins with dual role and can be involved in multidrug resistance, quorum‐quenching, sporulation and degradation of aromatic compounds, among other functions (Molina‐Henares *et al*., 2006). The *ohpR* and *ohpT* genes were highly and specifically induced by the presence of 2‐HPA (Fig. 2), strongly indicating that both genes play a role in 2‐HPA catabolism. Concerning control of *ohpA* gene expression, two upstream sequences might be putatively involved: the *ohpA* upstream sequence or, more probably, the *ohpT* upstream region considering that *ohpT* and *ohpA* genes would be part of the same transcriptional unit as suggested by two different bioinformatic algorithms for operon prediction based on intergenic distances (http://microbiome.wlu.ca/public/TUpredictions/Predictions/GCF_000203875.tus; Moreno‐Hagelsieb and Collado‐Vides, 2002) and comparative genomics (http://http://www.microbesonline.org/operons/gnc264198.html; Price *et al*., 2005). The operon structure composed by *ohpT* and *ohpA* genes was confirmed by quantitative real‐time PCR analysis of the extended intergenic region, including boundaries of both genes, that showed a specific induction pattern in presence of 2‐HPA (Fig. S2). On the contrary, the extended *ohpR‐ohpT* region was also assayed as a negative control showing no signal (Fig. S2). Consequently, to evaluate the presence of a promoter triggering expression of the *ohpT*‐*ohpA* operon, a transcriptional fusion was constructed including the *ohpR* gene and the *ohpT* upstream sequence to drive the expression of *gfp* gene (*P_ohpT_‐*GFP; Fig. 1B). This construct was introduced in *C. pinatubonensis* JMP134 cells, and the relative levels of GFP fluorescence were recorded growing in different substrates. Results showed that 2‐HPA significantly increased *P_ohpT_*‐GFP activity (Fig. 5) and, on the contrary, PA or aromatics that are degraded through the homogentisate pathway (phenylalanine, tyrosine, or 3‐HPA or 4‐HPA isomers) did not provoke any induction (Fig. 5); strongly suggesting that *ohpR*‐*ohpT* intergenic sequence contains a promoter that is driving the expression of an *ohpT‐ohpA* operon. This promoter would be specifically responsive to 2‐HPA, and not to analogous compounds or shared intermediates of homogentisate pathway.

### The OhpA enzyme is a self‐sufficient cytochrome P450 that belongs to the CYP116B subfamily

**Fig. 6:**
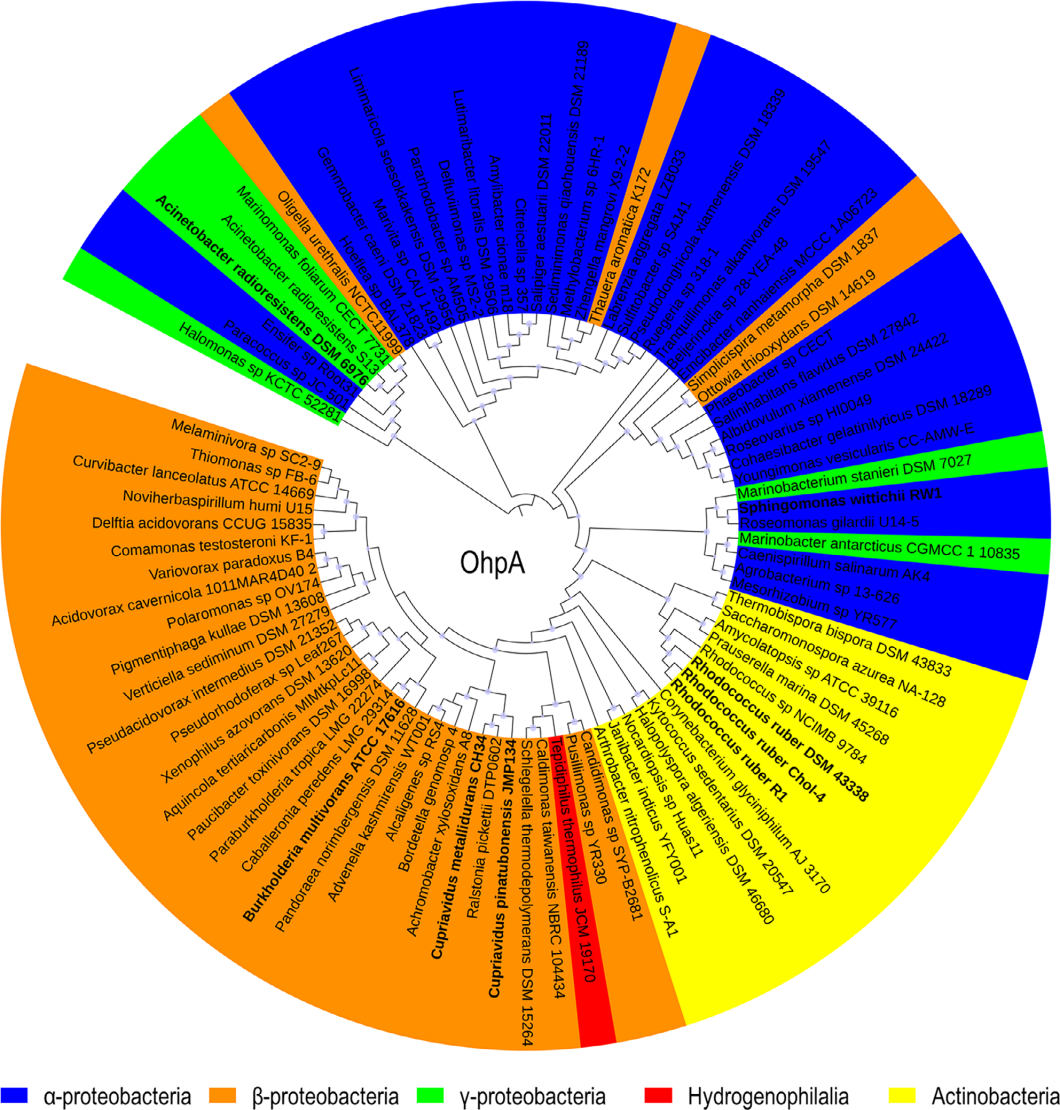
Evolutionary relationships among several OhpA homologues. Maximum likelihood topology provided by IQ‐TREE (Nguyen *et al*., 2015) based on sequence alignments calculated using MAFFT (Katoh *et al*., 2019) is shown with SH‐like approximate likelihood ratio support values (*n* = 1000) given at each node (values > 50% are shown). Sequences indicated in bold belong to strains tested by its ability to grow in 2‐HPA as a sole carbon and energy source. Blue, α‐proteobacteria; orange, β‐proteobacteria; green, γ‐proteobacteria; yellow, Actinobacteria; red, Hydrogenophilalia class.

**Fig. 7:**
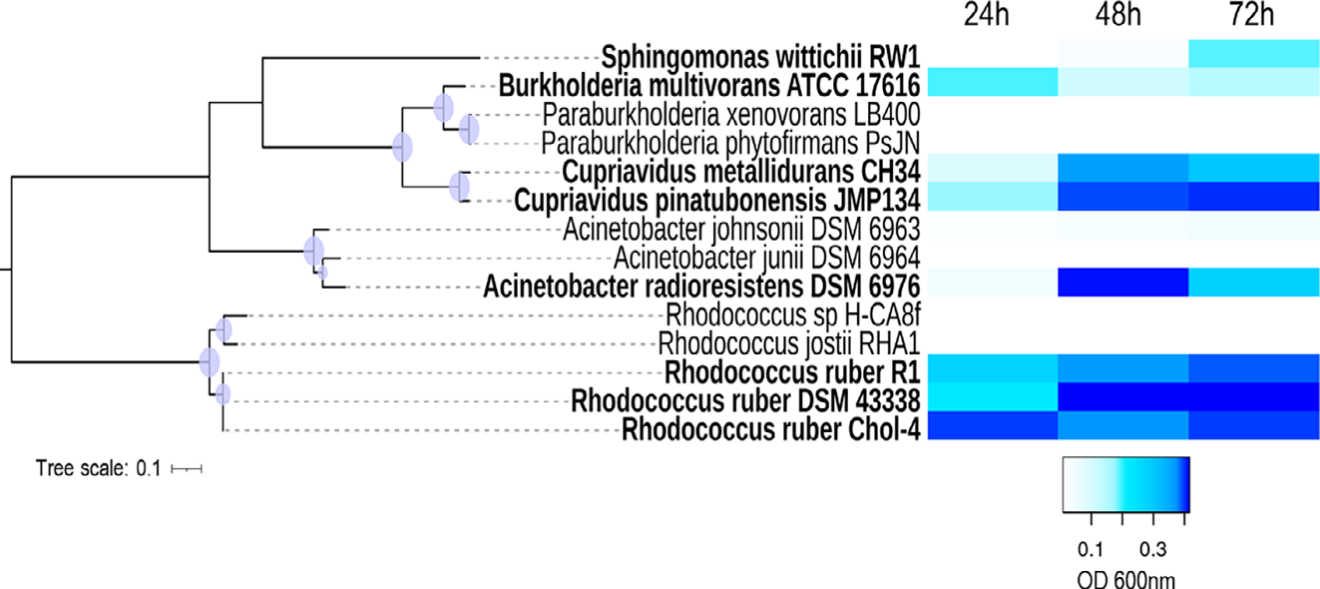
Growth on 2‐hydroxyphenylacetate (2‐HPA) of several bacterial species included in different taxonomic groups. Strains belonging to *Acinetobacter*, *Burkholderia*, *Cupriavidus*, *Paraburkholderia*, *Rhodococcus* and *Sphingomonas* genera were grown in mineral salt medium with 2.5 mM 2‐HPA as a sole carbon and energy sources. Bacterial strains that carry the *ohpA* gene are indicated in bold. Shading indicates optical density (OD) at 600 nm on 24, 48 and 72 h (average of two biological replicates). Lower OD at stationary phase is the result of cell clumping in some strains. A dendrogram based in GyrB sequences is shown as reference of phylogenetic relationship among close related 2‐HPA^+^ and 2‐HPA^−^ strains. The phylogenetic tree was constructed using IQ‐TREE (Nguyen *et al*., 2015) based on sequence alignments calculated utilizing MAFFT (Katoh *et al*., 2019). *Acinetobacter* species were supplemented with 0.25 mM tyrosine to overcome amino acid auxotrophy. The *S. wittichii* RW1 strain needed a previous adaptation in 2‐HPA to obtain reproducible growth.

A comparison of the amino acid sequence of OhpA (783 aa) with those of the UniProtKB/Swiss‐Prot database showed a 54% sequence identity between residues 19 to 443 with a non‐partner fused P450 enzyme CYP116 (also known as ThcB) from *Rhodococcus* sp. NI86/21, which is involved in degradation of herbicides S‐ethyl dipropylthiocarbamate (EPTC) and atrazine (Nagy *et al*., 1995a,b). This OhpA region contains cytochrome P450 cysteine haem‐iron ligand signature between residues 381 and 390 FGYGSHQCMG (PROSITE consensus pattern [F/W]‐[S/G/N/H]‐X‐[G/D]‐{F}‐[R/K/H/P/T]‐{P}‐C‐[L/I/V/M/F/A/P]‐[G/A/D]; Fig. 1C), indicative that N‐terminal region contains haem‐binding domain, and that residue C388 is expected to be the haem‐iron proximal (fifth) ligand (Werck‐Reichhart and Feyereisen, 2000; Denisov *et al*., 2005). Furthermore, residues 473‐771 of the C‐terminal portion of OhpA (Fig. 1C) shows 42% amino acid sequence identity to the phenoxybenzoate (POB) dioxygenase beta subunit (PDOR‐like) of *P. oleovorans*, which is the reductase subunit of POB dioxygenase that provides electrons to enable this enzyme to oxygenate 4‐carboxydiphenyl ether (Dehmel *et al*., 1995). This OhpA region comprises at least three key conserved functional parts (Fig. 1C), an FMN‐binding domain, a NAD(P)‐binding domain and a [2Fe‐2S] iron‐sulphur cluster‐binding domain, deduced by conserved domain analysis. Consequently, *ohpA* gene apparently encodes a self‐sufficient cytochrome P450 like the well‐known P450 RhF from *Rhodococcus* sp. NCIMB 9784, not requiring the involvement of additional proteins to transfer electrons from the reduced pyridine nucleotide to the active site and sharing a 66% amino acid identity on the overall sequence. A previous analysis of prokaryotic genome sequences using as bait P450 RhF from *Rhodococcus* sp. NCIMB 9784, permitted to find this novel kind of P450 redox system in *C. metallidurans* CH34, a close relative of *C. pinatubonensis* JMP134, allowing assignation of these *Cupriavidus* proteins as new members of the CYP116 family (CYP116B), founded by ThcB (non‐partner fused, CYP116A) from *Rhodococcus* sp. NI86/21 (De Mot and Parret, 2002), but whose self‐sufficient members lacks a natural substrate properly established. Based on similarity between haem domains of ThcB and *C. metallidurans* CH34 CYP116B1 protein, Warman *et al*. (2012) have tested this protein for activity over thiocarbamate herbicides, showing that EPTC and S‐propyl dipropyl diothiocarbamate were hydroxylated on propyl chains, proving thus that CYP116B1 has similar thiocarbamate‐oxydizing catalytic properties compared to the rhodococcal CYP116A1 protein, the P450 involved in oxidative degradation of EPTC (Nagy *et al*., 1995a). Notably, we found that *C. metallidurans* CH34 is also able to use 2‐HPA as a sole carbon and energy source (see below), and the amino acid sequence of strain CH34 CYP116B1 has 80% amino acid identity with strain JMP134 OhpA, strongly suggesting that natural substrate of this self‐sufficient cytochrome P450 CYP116B1‐like enzyme is 2‐HPA. A search for OhpA homologues in available bacterial genomes showed that this protein is present in diverse bacterial species mostly belonging to α‐ and β‐proteobacterial classes, and Actinobacteria phylum but also in a few members of the γ‐proteobacterial class (Fig. 6). It should be noted from examination of gene clusters of OhpA homologues that some Actinobacteria (*Arthrobacter nitrophenolicus*, *Janibacter indicus*, *Corynebacterium glyciniphilum*, *Saccharomonospora azurea* and *Thermobispora bispora*), α‐proteobacteria (*Caenispirillum salinarum*) and γ‐proteobacteria (*M. antarcticus* and *M. stanieri*) harbour the coding gene in close proximity to the *hmgA* gene (Fig. S3), strengthening the functional link among CYP116B subfamily members and the homogentisate pathway. We tested the capacity to grow on 2‐HPA in selected groups of close related strains, containing or not an OhpA homologue, across several taxonomic lineages (Fig. 7), that is *Acinetobacter, Burkholderia, Cupriavidus, Paraburkholderia, Rhodococcus and Sphingomonas genera*, revealing a perfect match among the presence of *ohpA* gene and the ability to use this compound as sole carbon and energy source (Fig. 7). Notably, the gene coding for the OhpA homologue (locus tag Bmul_5958, previously BMULJ_05568) harboured by the 2‐HPA‐degrading *Burkholderia multivorans* ATCC 17616 strain (Fig. 7) is induced in soil environment as revealed by *in vivo* expression technology (Nishiyama *et al*., 2010, see Table 2 in the reference), suggesting that the substrate of CYP116B1 homologues should be a natural carbon source in such habitat. The case of the 2‐HPA‐degrading *A. radioresistens* DSM 6976 strain (Fig. 7) is also particularly interesting since its OhpA homologue is 98% identical to the one described in *A. radioresistens* S13 as responsible for growth on alkanes (Minerdi *et al*., 2015). We consider unlikely that CYP116B1 homologues of some strains used in this work, such as those harboured by *Cupriavidus* species, accomplish such role in addition to 2‐HPA biodegradation, since neither JMP134 nor CH34 strains are able to use alkanes as growth substrates (data not shown).

Crystal structures of OhpA homologues from *A. radioresistens* S13 and *Tepidiphilus thermophiles* JCM 19170 have been recently solved, providing first bases for the structure‐guided design of new biocatalysts with these enzymes (Tavanti *et al*., 2018; Ciaramella *et al*., 2019; Zhang *et al*., 2020). According to the X‐ray structure of the homologue harboured by strain JCM 19170, it was revealed that the catalytic pocket of the enzyme is surrounded by 18 residues organized in a 4‐tiered system above the haem cofactor that are well conserved among CYP116B members (Tavanti *et al*., 2018). A sequence alignment analysis using all homologues of OhpA from Fig. 6, showed a well‐conserved catalytic pocket (Table S1) and haem‐binding domain (Table S2), suggesting a similar substrate profile among analysed strains. Nevertheless, in the case of OhpA homologue from *T. thermophiles,* a few non‐conservative substitutions in comparison with the consensus could reflect a different substrate preference (Tables S1 and S2; Tavanti *et al*., 2018). Consequently, enzyme assays of the self‐sufficient cytochrome P450 in *T. thermophiles* and *A. radioresistens* strains using 2‐HPA as substrate will provide additional clarification on the physiological substrate of such members of CYP116B subfamily.

## Conclusion

The results of this study provide evidence for the thus far unknown function of a self‐sufficient cytochrome P450, belonging to the CYP116B family. The 2‐HPA 5‐hydroxylase activity proposed here is in line with the recent report of proficient 5‐hydroxylation on the phenylacetic acid moiety of the synthetic anti‐inflammatory drug diclofenac (2‐[2‐(2,6‐dichloroanilino)phenyl]acetic acid) by the founder member of the CYP116B family, the self‐sufficient cytochrome P450 RhF from *Rhodococcus* sp. NCIMB 9784 (Klenk *et al*., 2017). The identification of 2‐HPA as natural substrate of the CYP116B family will allow kinetic and protein engineering studies providing guidelines for improvement and implementation of biocatalytic P450 processes such as those required for production of oxyfunctionalized high‐value compounds in pharmaceutical and related industries (Urlacher and Girhard, 2019).

## Experimental procedures

### Bacterial strains, plasmids and growth conditions

Bacteria and plasmids used in this study are listed in Table 1. *C. pinatubonensis* JMP134 and *P. putida* KT2440, and its derivatives were grown at 30°C in mineral salts medium, supplemented with 5 mM 2‐HPA, 3‐HPA, 4‐HPA, PA, tyrosine, phenylalanine, or fructose, plus the appropriate antibiotics, kanamycin (Km; 50 μg ml^−1^) or gentamicin (Gm; 30 μg ml^−1^). Other strains tested for growth on 2‐HPA were grown at 30°C in mineral salts medium, supplemented with 2.5 mM 2‐HPA. *Escherichia coli* Mach1 (Invitrogen, Carlsbad, CA, USA) was grown at 37°C in Luria‐Bertani (LB) medium. Growth was measured by optical density at 600 nm (OD_600_) with a Synergy HTX multimode plate reader (BioTek, Winooski, VT, USA). At least three biological replicates were performed for each growth measurement.

**Table 1:** Bacterial strains and plasmids used in this study.

Strain or plasmid	Relevant phenotype and/or genotype	Reference or source
*C. pinatubonensis* strains		
JMP134	2‐HPA^+^, 3‐HPA^+^, 4‐HPA^+^, PA^+^, tyrosine^+,^ phenylalanine^+^, fructose^+^, arabinose^‐^	DSMZ^a^
JMP134d*ohpA*	2‐HPA^−^, fructose^+^	This study
JMP134d*hmgA*	2‐HPA^−^, fructose^+^	This study
Other strains		
*P. putida* KT2440	2‐HPA^−^, arabinose^‐^	Bagdasarian *et al*. (1981)
*E. coli* Mach1	Δ*recA1398 endA1 tonA ϕ80ΔlacM15 ΔlacX74 hsdR(rK− mK+)*	Invitrogen, Carlsbad, USA
*R. ruber* Chol‐4	*ohpA* homologue, locus tag D092_RS23190	DSMZ
*R. ruber* DSM 43338	*ohpA* homologue, locus tag RR3_RS22805	DSMZ
*R. ruber* R1	*ohpA* homologue, locus tag E2561_01140	Farkas *et al*. (2020)
*R. jostii* RHA1	No *ohpA* homologue	McLeod *et al*. (2006)
*Rhodococcus* sp. H‐CA8f	No *ohpA* homologue	Undabarrena *et al*. (2018)
*C. metallidurans* CH34	*ohpA* homologue, locus tag RMET_RS25305	DSMZ
*B. multivorans* ATCC 17616	*ohpA* homologue, locus tag Bmul_5958	Nishiyama *et al*. (2010)
*P. xenovorans* LB400	No *ohpA* homologue	Chain *et al*. (2006)
*P. phytofirmans* PsJN	No *ohpA* homologue	Weilharter *et al*. (2011)
*A. radioresistens* DSM 6976	*ohpA* homologue, locus tag ACRAD_RS10630	DSMZ
*A. johnsonii* DSM 6963	No *ohpA* homologue	DSMZ
*A. juniii* DSM 6964	No *ohpA* homologue	DSMZ
*S. wittichii* RW1	*ohpA* homologue, locus tag SWIT_RS15645	DSMZ
Plasmids		
pCR2.1‐TOPO	Suicide vector in *C. pinatubonensis* JMP134, Km^R^	Invitrogen, Carlsbad, USA
pCR2.1‐d*ohpA*	pCR2.1 derivative containing internal fragment of *ohpA* gene, Km^R^	This study
pCR2.1‐d*hmgA*	pCR2.1 derivative containing internal fragment of *hmgA* gene, Km^R^	This study
pBS1	Broad host range vector, *araC*‐P_BAD_, Gm^R^	Bronstein *et al*. (2005)
pBS1‐*ohpA*	pBS1 derivative expressing *ohpA* gene, Gm^R^	This study
pBS1‐*hmgA*	pBS1 derivative expressing *hmgA* gene, Gm^R^	This study
pSEVA237M	Broad host range *msf*GFP reporter vector, Km^R^	Svenningsen *et al*. (2015)
P_ohpT_‐GFP	pSEVA237M derivative, *msf*GFP gene under control of *ohpT* promoter, Km^R^	This study

^a^ DSMZ: Deutsche Sammlung von Mikroorganismen und Zellkulturen GmbH (Braunschweig, Germany).

### Chromosomal disruption of gene sequences in *C. pinatubonensis* JMP134

Internal fragments of *ohpA* and *hmgA* were amplified by PCR using primer pairs listed in Table 2. The PCR products were cloned using the pCR2.1‐TOPO system (Invitrogen, Carlsbad, CA, USA) to generate plasmids listed in Table 1. For gene inactivation, suicidal pCR2.1‐d*ohpA* or pCR2.1‐d*hmgA* plasmids (Table 1) were electroporated in *C. pinatubonensis* JMP134 cells to get a one‐recombination‐event disruption of the target gene resulting in insertional mutants (Table 1), which were selected on LB agar containing 50 μg ml^−1^ Km. Correct insertions in all mutants were confirmed by PCR using primer pairs located in genomic DNA and suicidal plasmid DNA (Table 2), and subsequent sequencing.

**Table 2:** Primer pairs used in this study.

Purpose	Forward		Reverse	
	Name	Sequence (5′→3′)	Name	Sequence (5′→3′)
Gene inactivation	hmgA‐intFw	CTACGCCAACGGATTTCATC	hmgA‐intRv	CACAGATTGCCCTGGAACTT
	ohpA‐intFw	GACCAGATGCTGTGGGAAGT	ohpA‐intRv	CATCATCATCGAATGCAGGT
Real‐Time PCR analysis	ohpA‐Fw	CTGGGGATCGACTACGAGAT	ohpA‐Rv	CCGCCTTCTTCCTTGATGTA
	ohpT‐Fw	AGGAATTCATCGCGCAGAC	ohpT‐Fw	TCGACCTTCAGCAATTCCAT
	ohpR‐Fw	ACCTGCTTTATCGCCGTCAT	ohpR‐Fw	GTCGAGCAATCCCAGAAAGA
	hmgA‐Fw	ATCCGACCATGAACCTTACG	hmgA‐Rv	CTCAGTGGCGAACTCGTTG
	16S‐Fw	AGCGGTGGATGATGTGGATTA	16S‐Rv	TTGTCACCGGCAGTCTCTCTAG
Promoter fusion	p‐ohpT‐Fw	TAAGCAGGTACCCAGCACAGCGTACCGAATC	p‐ohpT‐Rv	TAAGCATCTAGATTGTCTCCGGTGTTGTGGTA
Expression constructs	ohpA1	TTGGGCTAGCAATTCCTGCCAGCCGTTGTCCACATTG	ohpA2	GTAATACGACTCACTATAGGGTCGTCATCAGCCTTGCT
	hmgA1	TTGGGCTAGCAATTCCTGCTTGACACGTCCATTGC	hmgA2	GTAATACGACTCACTATAGGACTCCGGAAAATGTCT
	pBS1a	GCAGGAATTCGCTAGCCCAA	pBS1b	CCTATAGTGAGTCGTATTAC

### Detection of transcripts by quantitative real‐time PCR

Cells of *C. pinatubonensis* JMP134 were grown on 5 mM 2‐HPA, 3‐HPA, 4‐HPA, PA or fructose as sole carbon and energy sources. Then, total RNA was obtained from 4 ml of mid‐log‐phase cells, using RNAprotect bacterial reagent and the RNeasy minikit (Qiagen, Chatsworth, CA, USA). The RNA was quantified using an Eon microplate spectrophotometer (BioTek, Winooski, VT, USA) and treated with the Turbo DNase kit (Ambion, Austin, TX, USA) to remove DNA contamination. The reverse transcription PCR was performed using the ImProm‐II reverse transcription system (Promega Corporation, Madison, WI, USA) with 1 μg of RNA in 20‐μl reaction mixtures. Real‐time PCR was performed using the Brilliant II SYBR Green QPCR Master Mix (Agilent Technologies, Santa Clara, CA, USA) and AriaMx Real‐time PCR System (Agilent Technologies). The PCR mixture (15 μl) contained 3.0 μl of template cDNA (diluted 1:10) and 0.2 μM (each) primer. Amplification was performed under the following conditions: 95°C for 5 min, followed by 40 cycles of 95°C for 30 s, 60°C for 30 s, and 72°C for 40 s, and finishing with a melting cycle from 55 to 95°C. Relative gene expression values were calculated using the comparative cycle threshold method (also known as the 2^−ΔΔ^ *^CT^* method; Schmittgen and Livak, 2008). 16S rRNA gene sequence (Reut_AR0020) was used as a reference gene (internal control) in these assays. Gene expression levels were normalized to the average value of the gene expression levels determined in the fructose treatment. Genes analysed by quantitative real‐time PCR were *ohpA* (Reut_B5278), *ohpR* (Reut_B5276), *ohpT* (Reut_B5277) and *hmgA* (Reut_B3923). Experiments were done in three biological replicates.

### Construction of plasmid derivatives expressing *ohpA* and *hmgA* genes

To obtain pBS1‐*ohpA* and pBS1‐*hmgA* plasmids (Table 1), which contain the *ohpA* and *hmgA* genes under the control of a L‐arabinose‐inducible promoter, the Gibson *et al*. (2009) assembly method was used. In brief, PCR products comprising *ohpA*, or *hmgA*, and pBS1 plasmid, were generated using primer pairs ohpA1‐ohpA2, hmgA1‐hmgA2 and pBS1a‐pbS1b, respectively, Table 2. These primers contain a 20‐bp terminal sequence homologous to the terminus of the fragment to be linked, and the sequences were combined and ligated to generate a new DNA molecule in a one‐step isothermal reaction (Gibson *et al*., 2009). Plasmid pBS1 derivatives were electroporated in strains JMP134d*ohpA*, JMP134d*hmgA* or *P. putida* KT2440 and selected in LB medium plus Gm (30 μg ml^−1^), or Km (50 μg ml^−1^), as appropriate. For expression of *ohpA* or *hmgA* genes driven by the heterologous P_BAD_ promoter, these derivatives were exposed to 5 mM l‐arabinose. All plasmid constructs obtained by the Gibson *et al*. (2009) assembly method, were confirmed by DNA sequencing.

### NADH oxidation assay in cells extracts

The substrate‐dependent oxidation of NADH was used to test specificity of OhpA in cell extracts of *P. putida* KT2440. The preparation of cell extracts in this bacterium has been previously described (Pérez‐Pantoja *et al*., 2009, 2000). Briefly, *P. putida* KT2440 and a derivative harbouring the pBS1‐ohpA construct were grown to late exponential phase in mineral medium with succinate (30 mM) as sole carbon source in the presence of 5 mM l‐arabinose plus antibiotic Gm when required. Then, cells were harvested, centrifuged at 8500 rpm for 10 min, washed twice with 1 vol. Tris/acetate (50 mM, pH 7.5) and suspended in 1 ml Tris/acetate (50 mM, pH 7.5) to be subjected to lysis by sonication with three pulses of 10 s at potency level 15 using an ultrasonic cell disruptor (Microson XL2000; Misonic Inc., Farmingdale, NY, USA). The lysates were subjected to two successive centrifugations at 16 000 rpm for 45 min at 4°C. Finally, supernatants were collected and used for enzyme assays. The substrate‐dependent oxidation of NADH was measured spectrophotometrically by the decrease of cofactor NADH at 340 nm (ε_340_ = 6300 M^−1^ cm^−1^) in a diode‐array Hewlett Packard HP 8452‐A UV/Vis spectrophotometer. The reaction was performed in 1.0 ml quartz cuvettes with a 1 cm light path and the standard assay mixture contained (ml^−1^) 35 mM Tris/acetate buffer (pH 7.5), 100 μM substrate (2‐HPA, 3‐HPA or 4‐HPA), 1 µM FAD, 0.2 mM NADH and a suitable quantity of cell extract. After non‐specific (in the absence of substrate) NADH oxidation at 340 nm was recorded for 2 min, each substrate was added and recording of NADH oxidation was continued for 2 min. Enzyme activity was calculated from the difference between the non‐specific and substrate‐dependent oxidation rates. One unit of enzyme activity was defined as the amount of crude extract that catalyses the oxidation of 1 µmol of NADH/min. The protein concentration in the cell extracts was estimated as described by Bradford (1976). Bovine serum albumin was used as protein standard.

### Construction of msfGFP reporter fusions

Putative promoter region was fused to the monomeric superfolder *msf*GFP gene of the pBBR1‐based broad‐host‐range vector pSEVA237M (Svenningsen *et al*., 2015) of the SEVA collection (Silva‐Rocha *et al*., 2013). PCR product comprising 1062 bp of the upstream region contiguous to translational start of the *ohpT* gene, also including *ohpR*, was obtained using primer pairs listed in Table 2. The amplified DNA fragments were cloned into the *Kpn*I‐*Xba*I restriction site of pSEVA237M, forming P‐*ohpT*‐*msf*GFP plasmid; transferred into strain JMP134 and selected in LB medium supplemented with 50 μg ml^−1^ Km. To evaluate promoter induction profiles, bacterial cells carrying promoter construction were grown overnight on LB medium, and then inoculated in cultures containing 5 mM 2‐HPA, 3‐HPA, 4‐HPA, PA, tyrosine, phenylalanine or fructose as the sole carbon and energy source, plus antibiotic Km. The cultures were incubated in a 96‐well microplate (Thermo Fisher Scientific, Rochester, NY, USA) at 30°C and the OD600 and the green fluorescence (excitation filter: 485/20, emission filter: 528/20) were measured in a Synergy HTX Multi‐Mode Reader (BioTek, Winooski, VT, USA).

### Analytical methods

The presence of 2‐HPA, 3‐HPA and 4‐HPA was determined by high‐performance liquid chromatography using cell‐free supernatants from resting cells grown on 20 mM fructose (JMP134 derivatives) or succinate 30 mM (KT2440 derivatives) plus 2.5 mM 2‐HPA or arabinose (as inducers of *ohpA* expression in JMP134 or KT2440 strains, respectively), washed twice with 1 volume of phosphate buffer (14 g l^−1^ Na_2_HPO_4_·12H_2_O, 2 g l^−1^ KH_2_PO_4_), and subsequently incubated with 1 mM 2‐HPA, 3‐HPA and 4‐HPA. Samples (2 ml) were obtained at different times, filtered (0.22 µm) and injected into a JASCO liquid chromatograph LC‐4000 (JASCO, Oklahoma City, OK, USA) equipped with a Kromasil 100‐3.5‐C18 4.6 mm diameter column. A methanol‐H_2_O (40:60) mixture containing 0.1% (vol/vol) phosphoric acid was used as the solvent, at a flow rate of 0.8 ml min^−1^. The column effluent was monitored at 210 nm for all compounds. Retention times for 2‐HPA, 3‐HPA and 4‐HPA were 12.1, 11.7 and 11.3 min, respectively.

### Bioinformatic tools

The *ohpA* gene sequences were retrieved from non‐redundant protein sequences database of GenBank (https://blast.ncbi.nlm.nih.gov/Blast.cgi; NCBI Resource Coordinators, 2018). Only proteins displaying at least 45% amino acid identity with the entire *ohpA* gene of *C. pinatubonensis* JMP134 were considered for analysis. Protein similarity and conserved domain searches were performed with the BLASTP program and the Conserved Domains Database from the NCBI website using default parameters (Johnson *et al*., 2008; Lu *et al*., 2020). Evolutionary relationships were inferred by IQ‐TREE web server tools (http://iqtree.cibiv.univie.ac.at/) designed for estimate maximum‐likelihood phylogenies (Nguyen *et al*., 2015) employing ModelFinder as model‐selection method (Kalyaanamoorthy *et al*., 2017) and UFBoot2 for ultrafast bootstrap approximation (Hoang *et al*., 2018) with the ‐m TEST, ‐bb 1000 and ‐alrt 1000 options. Sequence alignments for phylogenetic reconstruction were calculated with MAFFT online service (https://mafft.cbrc.jp/alignment/server/) using Auto (FFT‐NS‐1, FFT‐NS‐2, FFT‐NS‐i or L‐INS‐i; depends on data size) strategy (Katoh *et al*., 2019). Visualization and edition of phylogenetic trees were performed by the Interactive Tree Of Life (iTOL) online tool (https://itol.embl.de/; Letunic and Bork, 2019).

### Chemicals

2‐HPA, 3‐HPA, 4‐HPA, l‐phenylalanine and homogentisic acid were purchased from Sigma‐Aldrich (Steinheim, Germany). PA, l‐tyrosine, d(‐)‐fructose and l(+)‐arabinose were purchased from Merck (Darmstadt, Germany).

## Conflict of interest

Authors declare no conflict of interest.

## Supporting information

**Fig. S1.** Brown color, presumably pyomelanin derived from homogentisate, in liquid cultures of *Cupriavidus pinatubonensis* JMP134d*hmgA* derivative when exposed to 2‐HPA. (A) Growth of *C. pinatubonensis* wild type (JMP134), *hmgA* mutant (JMP134d*hmgA*) and *hmgA* mutant expressing *hmgA* gene driven by a heterologous P_BAD_ promoter (JMP134d*hmgA* pBS1‐*hmgA*) on 2‐HPA as sole carbon and energy sources, in the presence (+) or absence (−) of l‐arabinose after 8 days. Phenylacetate was used as a control because it is degraded employing a different catabolic pathway, non‐related to homogentisate production (Pérez‐Pantoja *et al*., 2008). It should be mentioned that in living cells homogentisate polymerizes spontaneously to form pyomelanin, a brown color polymer, at short incubation times (Schmaler‐Ripcke *et al*. 2009). (B) HPLC‐UV chromatogram of supernatant from resting cells of strain JMP134d*hmgA* grown on fructose, washed, and subsequently exposed to 1 mM 2‐HPA. Sample was obtained at 24 h, filtered (0.22 μm) and injected into a JASCO liquid chromatograph LC‐4000 (JASCO, Oklahoma City, OK, USA) equipped with a Kromasil 100‐3.5‐C18 4.6 mm diameter column. A methanol‐H_2_O (60:40) mixture containing 0.1% (vol/vol) phosphoric acid was used as the solvent, at a flow rate of 1 ml min^‐1^. The column effluent was monitored at 210 nm. Retention time for 2‐HPA was 5.6 min. A signal putatively related to homogentisate was found at retention time of 2.3 min. This signal has a UV‐Vis spectrum and retention time identical to that of the homogentisate analytical standard showed in (C).
**Fig. S2.** Transcript levels of putative operon formed by *ohpT* and *ohpA* genes from *Cupriavidus pinatubonensis* JMP134 cells exposed to 2‐HPA. Real‐time PCR analysis was performed for intergenic zones *ohp*R‐*ohp*T and *ohp*T‐*ohp*A, as indicated in Figure, in cells grown on 2‐HPA, 4‐HP4 or fructose (control) as a sole carbon and energy sources. Transcript levels were normalized to the average value of transcript levels in fructose treatment. Additionally, 16S rRNA levels were used as a reference gene (internal control). All experiments were performed in three biological replicates. Error bars represent SEM. Different letters indicate statistically significant differences between treatments for each gene (one‐way analysis of variance, *P* < 0.05; Tukey’s test, *P* < 0.05).
**Fig. S3.** Gene clusters in which homologues to the cytochrome P450‐encoding gene (*ohp*A) are found from different bacterial lineages. Phylogenetic tree of OhpA homologues was constructed using IQ‐TREE (Nguyen *et al*., 2015) based on sequence alignments calculated employing MAFFT (Katoh *et al*., 2019), and it is displayed with SH‐like approximate likelihood ratio support values (*n* = 1000) given at each node (values > 50% are shown). The putative functions of the different genes included in the clusters are indicated on the right. The sizes of genes are to scale. The numbers included in the OhpA‐encoding genes (black arrow) are indicating amino acid identity (%) with OhpA from *C. pinatubonensis* JMP134.
**Table S1.** Sequence alignment of the catalytic pocket in OhpA homologues using as reference CUB06540 from *Tepidiphilus thermophilus* JCM 19170.
**Table S2.** Sequence alignment of heme binding domain in OhpA homologues using as reference the protein from *Cupriavidus pinatubonensis* JMP134.
