# Supplementary material for: Identification of a self‐sufficient cytochrome P450 monooxygenase from Cupriavidus pinatubonensis JMP134 involved in 2‐hydroxyphenylacetic acid catabolism, via homogentisate pathway
Source: Microb Biotechnol. 2021 Jun 22;14(5):1944–60. doi: 10.1111/1751-7915.13865 (PMC8449657; doi:10.1111/1751-7915.13865)
Supplement: Supplementary file 1 — Fig. S1. Brown color, presumably pyomelanin derived from homogentisate, in liquid cultures of Cupriavidus pinatubonensis JMP134dhmgA derivative when exposed to 2‐HPA. (A) Growth of C. pinatubonensis wild type (JMP134), hmgA mutant (JMP134dhmgA) and hmgA mutant expressing hmgA gene driven by a heterologous PBAD promoter (JMP134dhmgA pBS1‐hmgA) on 2‐HPA as sole carbon and energy sources, in the presence (+) or absence (−) of l‐arabinose after 8 days. Phenylacetate was used as a control because it is degraded employing a different catabolic pathway, non‐related to homogentisate production (Pérez‐Pantoja et al., 2008). It should be mentioned that in living cells homogentisate polymerizes spontaneously to form pyomelanin, a brown color polymer, at short incubation times (Schmaler‐Ripcke et al. 2009). (B) HPLC‐UV chromatogram of supernatant from resting cells of strain JMP134dhmgA grown on fructose, washed, and subsequently exposed to 1 mM 2‐HPA. Sample was obtained at 24 h, filtered (0.22 μm) and injected into a JASCO liquid chromatograph LC‐4000 (JASCO, Oklahoma City, OK, USA) equipped with a Kromasil 100‐3.5‐C18 4.6 mm diameter column. A methanol‐H2O (60:40) mixture containing 0.1% (vol/vol) phosphoric acid was used as the solvent, at a flow rate of 1 ml min‐1. The column effluent was monitored at 210 nm. Retention time for 2‐HPA was 5.6 min. A signal putatively related to homogentisate was found at retention time of 2.3 min. This signal has a UV‐Vis spectrum and retention time identical to that of the homogentisate analytical standard showed in (C). Fig. S2. Transcript levels of putative operon formed by ohpT and ohpA genes from Cupriavidus pinatubonensis JMP134 cells exposed to 2‐HPA. Real‐time PCR analysis was performed for intergenic zones ohpR‐ohpT and ohpT‐ohpA, as indicated in Figure, in cells grown on 2‐HPA, 4‐HP4 or fructose (control) as a sole carbon and energy sources. Transcript levels were normalized to the average value of transcript levels in fructose tr [file MBT2-14-1944-s001.doc]

**SUPPLEMENTARY MATERIAL**

**Identification of a self-sufficient cytochrome P450 monooxygenase from *Cupriavidus pinatubonensis* JMP134 involved in 2-hydroxyphenylacetic acid catabolism, via homogentisate pathway**

Raúl A. Donoso^1^, Daniela Ruiz^2,3^, Carla Gárate-Castro^1,3^, Pamela Villegas^1^, José Eduardo González-Pastor^4^, Víctor de Lorenzo^5^, Bernardo González^2,3#^, and Danilo Pérez-Pantoja^1#^

**
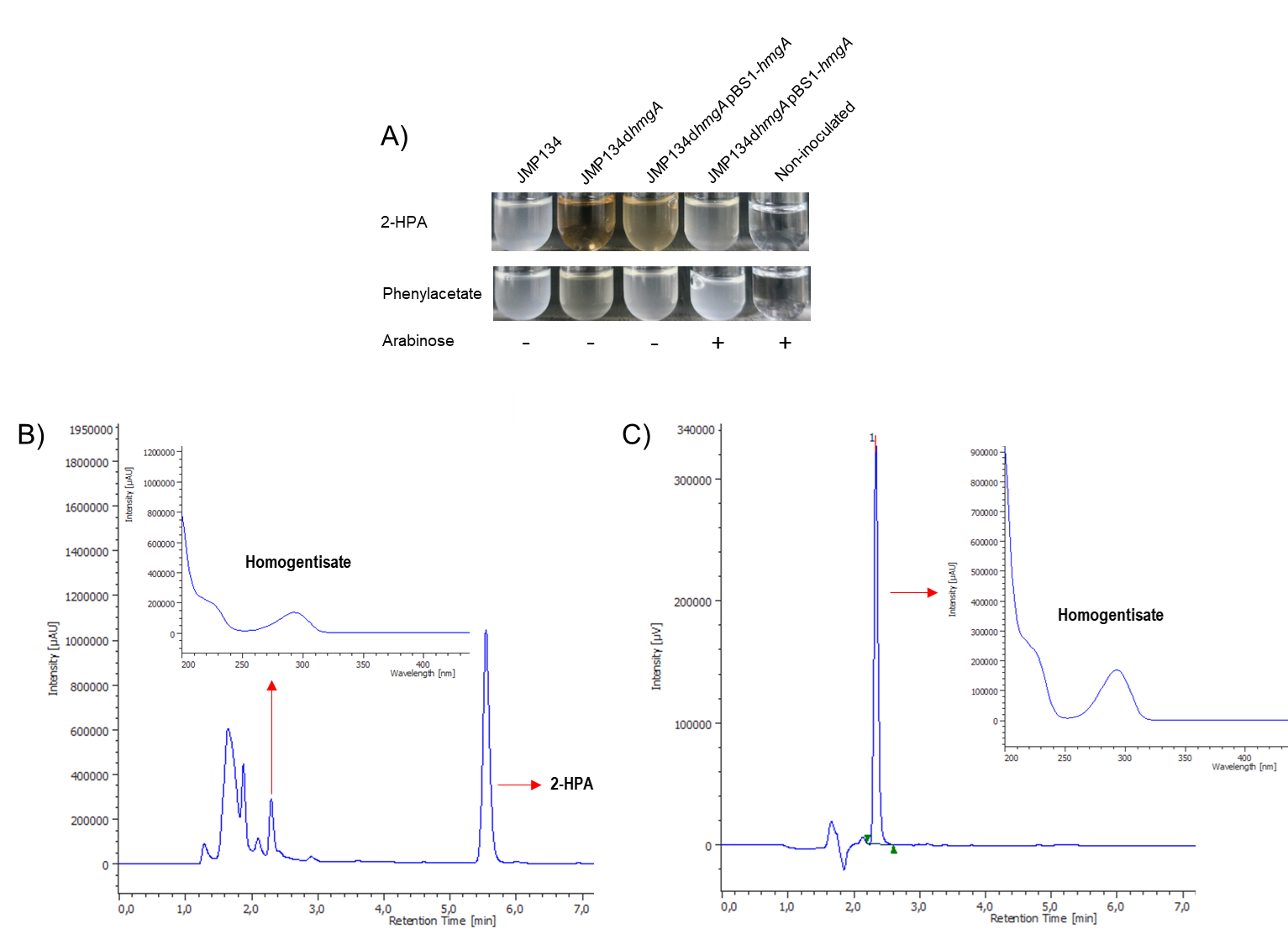
**

**Figure S1.** Brown color, presumably pyomelanin derived from homogentisate, in liquid cultures of *Cupriavidus pinatubonensis* JMP134d*hmgA* derivative when exposed to 2-HPA. A) Growth of *C. pinatubonensis* wild type (JMP134), *hmgA* mutant (JMP134d*hmgA*) and *hmgA* mutant expressing *hmgA* gene driven by a heterologous P_BAD_ promoter (JMP134d*hmgA* pBS1-*hmgA*) on 2-HPA as sole carbon and energy sources, in the presence (+) or absence (-) of L-arabinose after 8 days. Phenylacetate was used as a control because it is degraded employing a different catabolic pathway, non-related to homogentisate production (Pérez-Pantoja *et al*., 2008). It should be mentioned that in living cells homogentisate polymerizes spontaneously to form pyomelanin, a brown color polymer, at short incubation times (Schmaler-Ripcke *et al.* 2009). B) HPLC-UV chromatogram of supernatant from resting cells of strain JMP134d*hmgA* grown on fructose, washed, and subsequently exposed to 1 mM 2-HPA. Sample was obtained at 24 h, filtered (0.22 μm) and injected into a JASCO liquid chromatograph LC-4000 (JASCO, Oklahoma City, OK, USA) equipped with a Kromasil 100-3.5-C18 4.6 mm diameter column. A methanol-H_2_O (60:40) mixture containing 0.1% (vol/vol) phosphoric acid was used as the solvent, at a flow rate of 1 ml min^-1^. The column effluent was monitored at 210 nm. Retention time for 2-HPA was 5.6 min. A signal putatively related to homogentisate was found at retention time of 2.3 min. This signal has a UV-Vis spectrum and retention time identical to that of the homogentisate analytical standard showed in C).

**References**

Pérez-Pantoja, D., De la Iglesia, R., Pieper, D.H., and González, B. (2008) Metabolic reconstruction of aromatic compounds degradation from the genome of the amazing pollutant-degrading bacterium *Cupriavidus necator* JMP134. FEMS Microbiol Rev 32:736–794.

Schmaler-Ripcke, J., Sugareva, V., Gebhardt, P., Winkler, R., Kniemeyer, O., et al. (2009) Production of pyomelanin, a second type of melanin, via the tyrosine degradation pathway in *Aspergillus fumigatus*. Appl. Environ. Microbiol. 75:493-503.

**
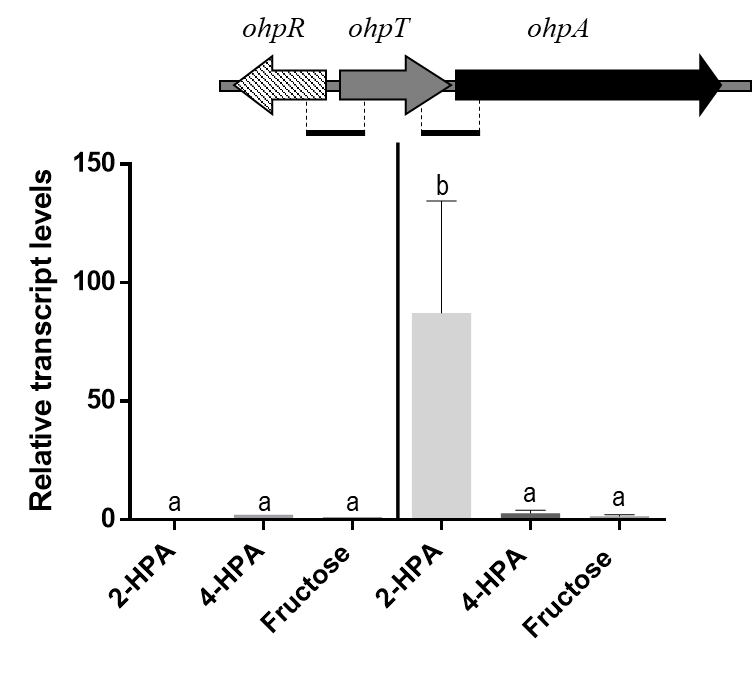
**

**Figure S2.** Transcript levels of putative operon formed by *ohpT* and *ohpA* genes from *Cupriavidus pinatubonensis* JMP134 cells exposed to 2-HPA. Real-time PCR analysis was performed for intergenic zones *ohp*R-*ohp*T and *ohp*T-*ohp*A, as indicated in Figure, in cells grown on 2-HPA, 4-HP4 or fructose (control) as a sole carbon and energy sources. Transcript levels were normalized to the average value of transcript levels in fructose treatment. Additionally, 16S rRNA levels were used as a reference gene (internal control). All experiments were performed in three biological replicates. Error bars represent SEM. Different letters indicate statistically significant differences between treatments for each gene (one-way analysis of variance, P < 0.05; Tukey’s test, P < 0.05).


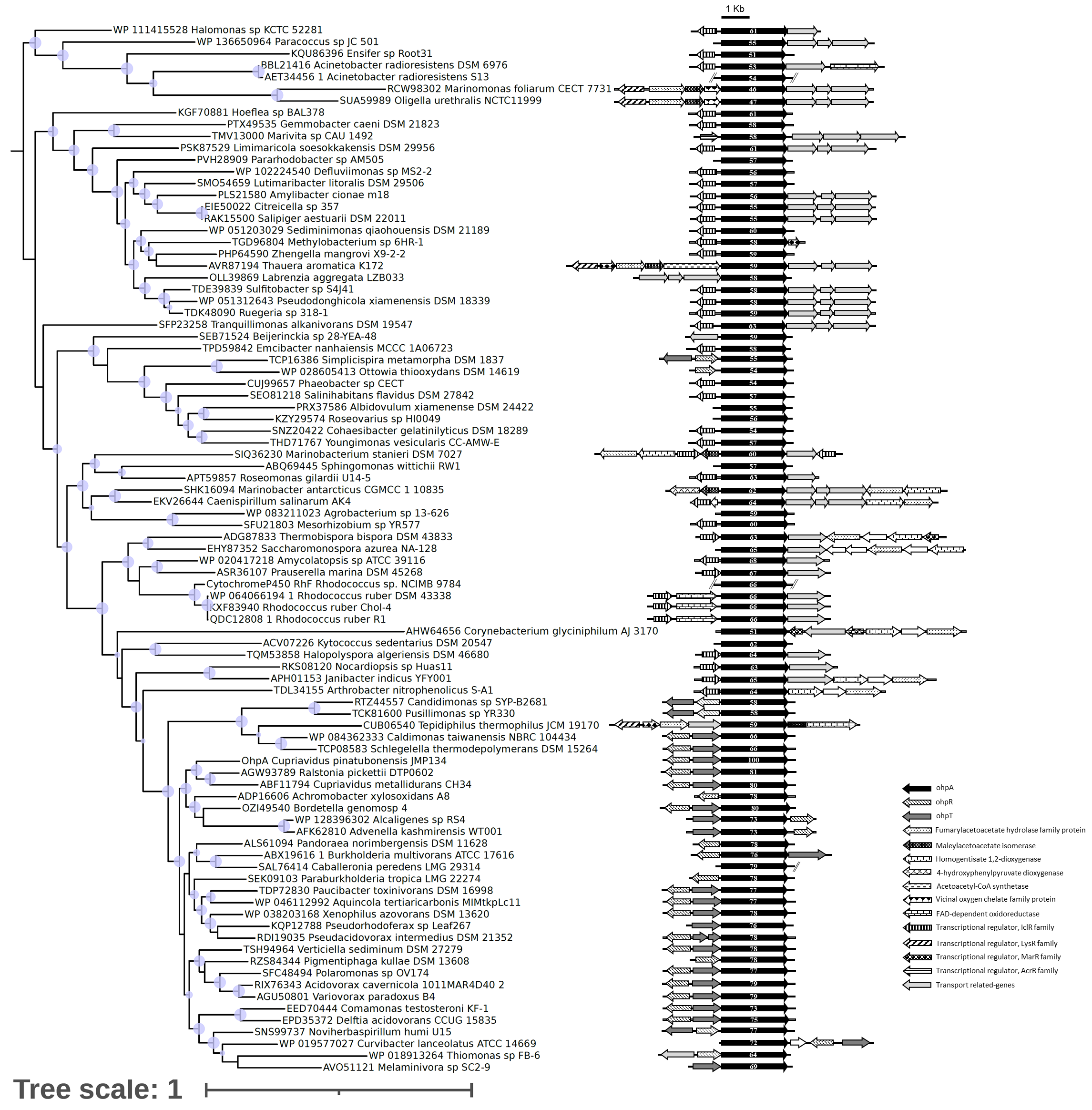


**Figure S3**. Gene clusters in which homologues to the cytochrome P450-encoding gene (*ohp*A) are found from different bacterial lineages. Phylogenetic tree of OhpA homologues was constructed using IQ-TREE (Nguyen *et al*., 2015) based on sequence alignments calculated employing MAFFT (Katoh *et al*., 2019), and it is displayed with SH-like approximate likelihood ratio support values (n=1000) given at each node (values >50% are shown). The putative functions of the different genes included in the clusters are indicated on the right. The sizes of genes are to scale. The numbers included in the OhpA-encoding genes (black arrow) are indicating amino acid identity (%) with OhpA from C*. pinatubonensis* JMP134.

**References**

Nguyen, L.T., Schmidt, H.A., von Haeseler, A., and Minh, B.Q. (2015) IQ-TREE: a fast and effective stochastic algorithm for estimating maximum-likelihood phylogenies. Mol Biol Evol 32:268-74.

Katoh, K., Rozewicki, J., and Yamada, K.D. (2019) MAFFT online service: multiple sequence alignment, interactive sequence choice and visualization. Brief Bioinform 20:1160-1166.


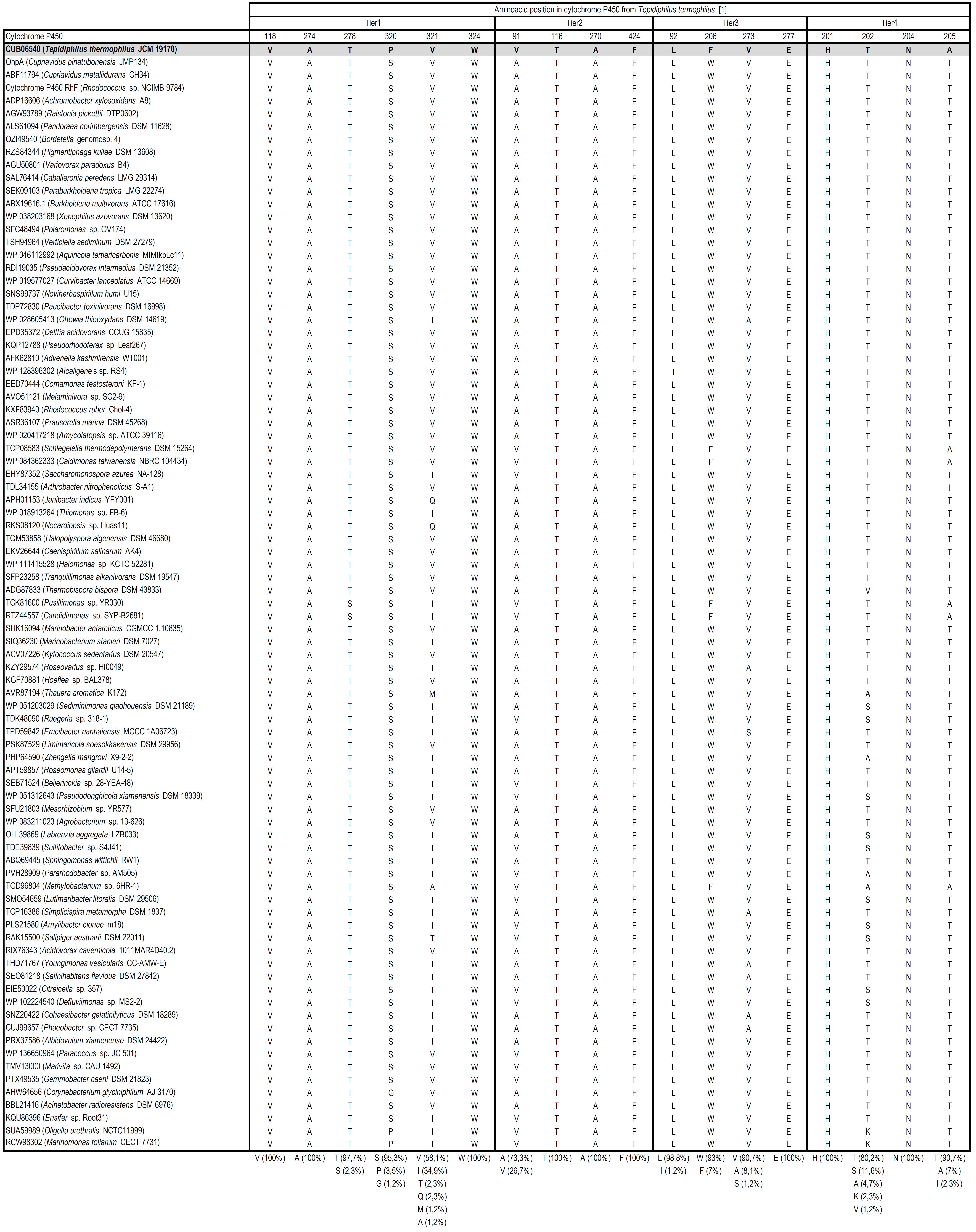


**Table S1.** Sequence alignment of the catalytic pocket in OhpA homologues using as reference CUB06540 from *Tepidiphilus thermophilus* JCM 19170 (highlighted in gray).

[1] Tavanti M., *et al*. (2018) The crystal structure of P450-TT heme-domain provides the first structural insights into the versatile class VII P450s. Biochem Biophys Res Commun 501:846-850.


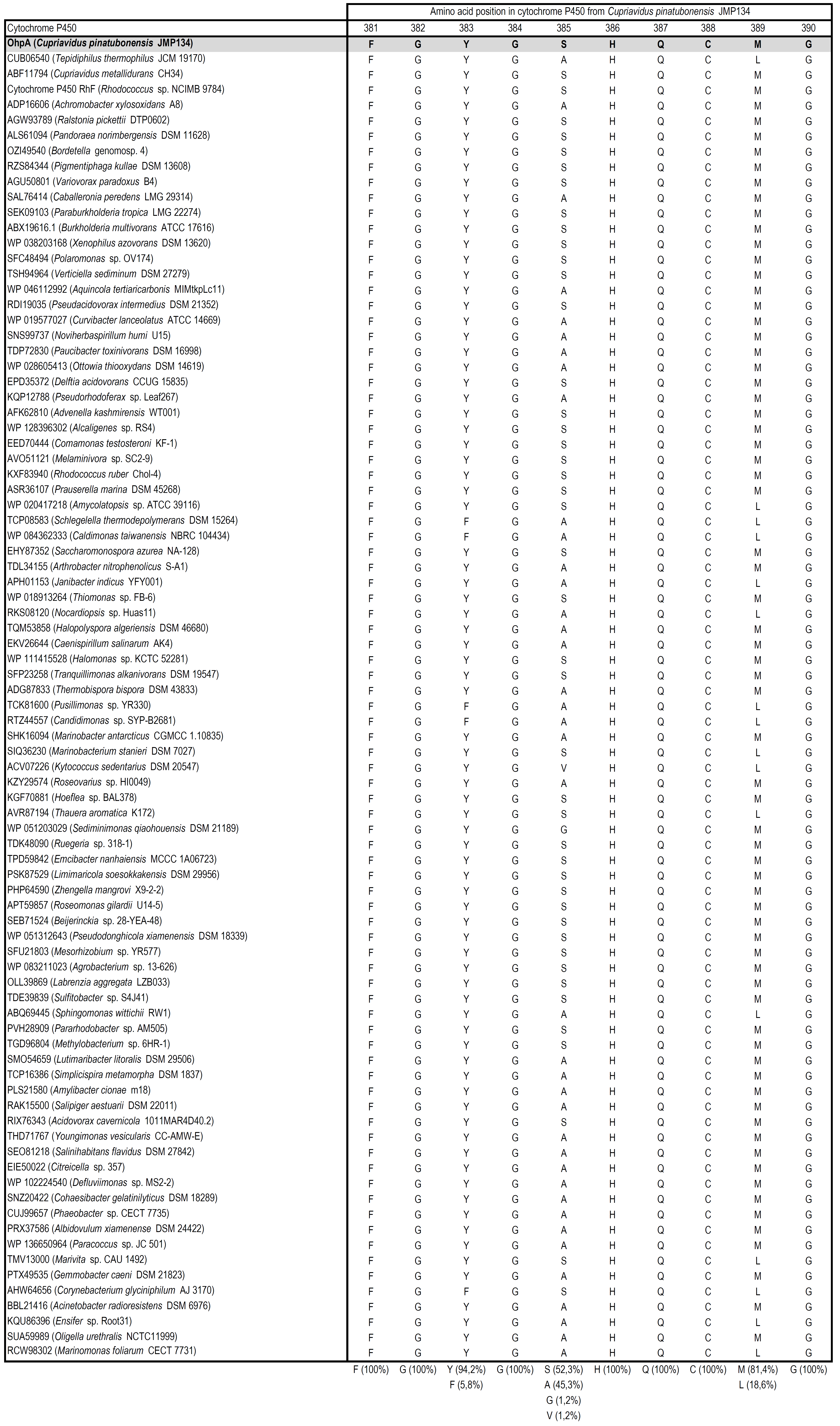


**Table S2.** Sequence alignment of heme binding domain in OhpA homologues using as reference the protein from *Cupriavidus pinatubonensis* JMP134 (highlighted in gray).
